# Review of medical professional organizations in developed countries: problems of decentralized membership registers

**DOI:** 10.3934/publichealth.2019.4.437

**Published:** 2019-10-22

**Authors:** M Carmen Bautista, Beatriz G Lopez-Valcarcel

**Affiliations:** 1 Medical council of Las Palmas, Spain and University of Las Palmas de Gran Canaria, Las Palmas de Gran Canaria, Spain; 2 Dept. Quantitative Methods for Economics and Management University of Las Palmas de Gran Canaria, Las Palmas de Gran Canaria, Spain

**Keywords:** developed countries, professionalism, physicians, medical councils, membership registers, professional regulation, decentralization, quality of regulation

## Abstract

This article provides a critical review of international experiences regarding the professional organization of physicians and the registration of doctors in developed countries. The problems faced by professional medical organizations in the EU-15 countries, Japan, the United States and Canada, are examined. Medical professional groups differ in several dimensions, including obligatory registration versus voluntary membership or types of registration (centralized, indirect, or delegated). The centralization-decentralization axis is a key aspect for the analysis. While decentralized systems are better able to adapt to the idiosyncrasy of a particular region, decentralization is identified as a source of potential problems in the organization of medical doctors. Some of these problems (discrepancies in positions on health matters, problems with the reliability of statistical information on medical demography at national level, deficient mechanisms for the control of doctors who have lost their licenses) might have consequences for the quality of the health care system.

## 1. Introduction

It is a matter of debate as to whether doctors should regulate themselves or not [1]–[9] and how this regulation affects the quality of the health system [10]–[14] and indeed the quality of life of citizens. Among those in favor of self-regulation, there is additional disagreement as to whether or not regulation should be delegated to corporate entities subject to public law [15]–[17]. And when a country chooses to delegate regulation to medical councils, there is a third level of discussion, which is the subject of this study, whether the registration and licensing of doctors is and should be centralized nationally, or, on the contrary, be delegated to local bodies. This article examines the registration process for the EU-15 countries, Japan, the United States and Canada, and identifies decentralization as a source of serious problems in the organization of medical doctors. A previous study [18] reviewed five dimensions identified for describing the registration and licensing of physicians in the EU, including governance and regulatory bodies, but the centralization/decentralization issue is only addressed descriptively and marginally. As far as we know this is the first international study of the subject.

This study covers a gap in the literature on medical regulation and licensing in developed countries because the centralization-decentralization debate has not been addressed so far. It is relevant for the area of public health, particularly for those responsible for the regulation of the medical profession and for the medical associations and councils as the deficiencies found and discussed in the article could orientate changes to improve their performance.

Specifically, this article points to the risks associated to the decentralization of the medical registries at subnational levels (states, provinces or regions). Some of them, as the effective control of the sanctioned physicians, are serious enough to call the attention of regulators.

## 2. Materials and methods

We started by selecting 18 countries representing the developed Western world, the EU-15 and United States of America, Canada and Japan. We then performed an on line exhaustive search of medical organizations in the selected countries. Each country has a different configuration and many medical organizations with different duties and competences (academic, scientific, regulatory). The inclusion criteria for selecting an organization for the study is that a) it is a professional organization of doctors; b) it has some competences in regulation of the profession and/or in the registration of physicians. We have included a glossary at the end of the text.

Once identified the organizations, we downloaded in a systematic way the information contained in their official webpages. The template for gathering the information contained the following items: year of creation, official competences (accreditation, deontologic, expedition of drug prescriptions, medical responsibility insurance, health campaigns to the population,…), other competences; if those competences are exclusive or shared with other/s organism/s; degree of decentralization and type (geographic or by medica, specialties); Compulsory/Volontary affiliation; requirements of access; economic-finance management (autonomy, sources of funding including subsidies, distribution of incomes, budget); are there any vinculated organitations or societies (foundations, insurance companies, banks); Does it belong to international organizations? Which ones?; communication channels (own or external; use of social networs; channels for communication with the members); existence of periodical publications, periodicity, impact).

That information has been analyzed according to a conceptual framework based on three dimensions, legal status, type of registration and degree of (de)centralization of licensing. These dimensions have been identified as crucial in light of the information gathered and the personal experience of the main author as a manager of a medical council. As we conclude that out of all the variables considered, the most relevant is centralization/decentralization, we then discuss the advantages and shortcomings of the decentralization.

## 3. Results

### 3.1. Classification of medical associations: public law corporations or independent professional associations

There is no standard way to classify medical professional groups. One option is to classify them by their regulatory body, whether the government itself, a government agency, or a corporation subject to public law [17]. Closely related to this topic is the question about who is in charge of registration and licensing of medical doctors, health authorization offices (often attached to public bodies such as the Ministry of Health) or medical chambers [19].

We classify medical professional organizations by their legal identity, identifying two categories: 1. corporations governed by public law with a separate legal entity and 2. independent professional associations or federations.

Public law corporations (category 1) are those bodies to which the public administration in their country has delegated functions, which vary greatly from nation to nation. In contrast, independent professional associations / federations (category 2) are composed of a set of persons or associations (in the case of federations) united by professional affinity for the purpose of defending their interests.

An important practical implication of the classification concerns to registration: obligatory registration or voluntary membership. Doctors in countries with public law medical corporations are legally obligated to belong to these corporate groups in order to work. In contrast, membership in all the professional associations is voluntary. In those countries in which the registration of doctors is not delegated to a public law corporation, it is the government itself, through one or another public agency, which is responsible for the supervision of these professionals [5].

Table 1 classifies national medical professional organizations for the 18 countries studied. In some countries, like the United Kingdom, Ireland and Canada, there co-exist two kinds of organizations, both public law corporations and independent professional associations. In these cases, the responsibility for regulation and registration rests with the public law corporations. In the appendix is the list of organizations with their respective web addresses.

As can be seen in table 2 (English translation of the organization names), the names given these general councils or medical associations do not always match their legal status.

**Table 1.**
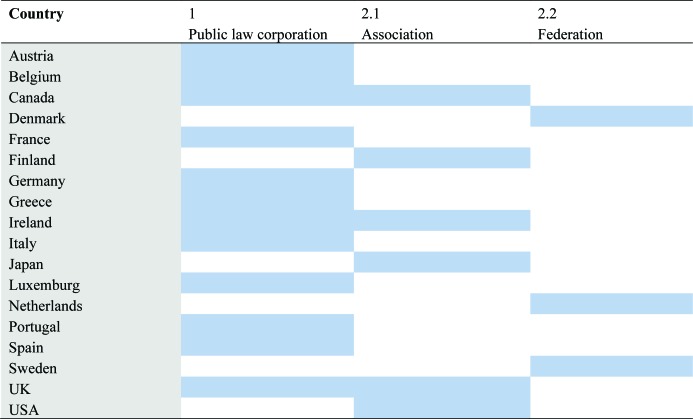
National medical professional organizations by legal identity.

**Table 2.**
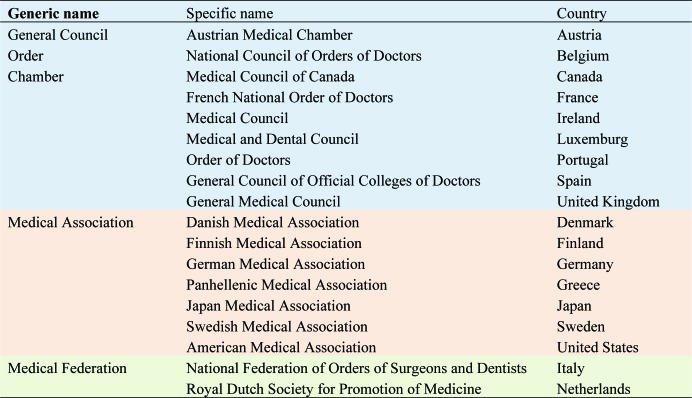
Classification of medical professional organizations by name.

### 3.2. Types of registration: centralized, indirect, and delegated

The licensing of doctors is a primary task both of public law corporations and the medical associations [19]. There are three types of registration: centralized, indirect, and delegated. In Table 3 we classify the national medical professional associations by type of registration, and we include also the number of offices (model 1, centralized registry), the number of delegated registries (model 2), and the number of associations that are responsible for registration in model 3 (indirect registries).

**Table 3.**
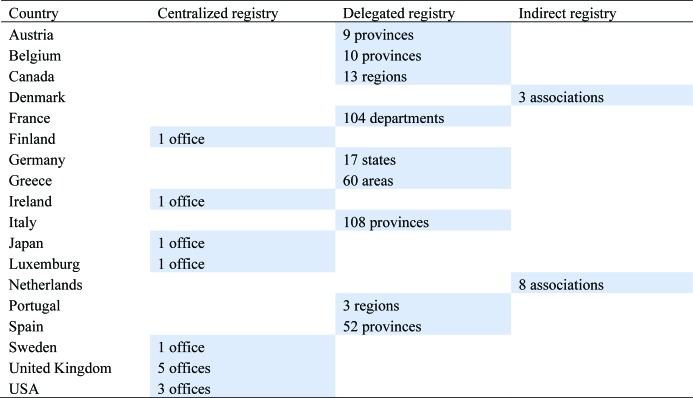
Classification of national medical professional organizations by type of registration.

This classification overlaps with that in table 1 (by type of organizations, public law corporations, associations and federations). Table 3 shows that in the three countries in which there are only independent medical associations (Finland, Japan and United States), registration is centralized in the association itself, irrespective of the number of physical offices in which it may take place. The three countries characterized by medical federations (Denmark, Netherlands and Sweden) have basically two types of registration. In Denmark and the Netherlands, registration is indirect. The doctor does not register directly with the federation, but rather in one of the federation's component associations. In Sweden, on the contrary, the doctor must register at the national level and then is registered in one of the one hundred associations of the federation. In most of the countries organized through public law corporations (Austria, Belgium, Canada, France, Germany, Greece, Italy, Portugal, Spain), governments have delegated registration to provincial or regional corporate bodies.

### 3.3. Decentralization or centralization

While the countries with public law corporations may or may not choose to decentralize the registry, those with professional associations all have central registries [Table 4].

**Table 4.** Typology by legal form and centralization of registry.

	Centralized	Decentralized
public law corporation	Ireland	Austria
	Luxemburg	Belgium
	United Kingdom	Canada
		France
		Germany
		Greece
		Italy
		Portugal
		Spain
professional association	Finland	-
	Denmark	
	Japan	
	Netherlands	
	Sweden	
	USA	

In countries without a central register, there is a wide variety in the level of autonomy of the geographical divisions, which in turn affects the map of regulation and verification of the medical profession in the country. The power of local organizations, which is related to the level of decentralization of financing, tasks and responsibilities from the national headquarters, is an aspect that differs among countries.

## 4. Discussion

Out of the many variables analyzed for each national medical association there are three main axes, legal status (public law corporations or independent professional associations), type of registry (centralized, indirect, and delegated) and centralization/decentralization of the doctor's registry. The later is associated to the main problems for the organization and the full medical system of the country.

The question on centralization/decentralization of the registry and licensing of doctors takes us to other more general issues as those on governance and standardization [20]. It is not only a question on who is responsible for gathering and distributing the information on active doctors, it has also to do with the management of medical organizations and the distribution of power. Technically it is feasible to design and share distributed registries, in fact medicine is an area very familiar with the use of shared registries based on medical records, treatment use, or clinical trials. The registry of doctors has a component of information, but it is beyond information as it involves licensing and the control of misconduct and inappropriate professional conduct. The organizations managing physician medical practice status, and the data and grounds for administrative sanctioning of physicians shows a large variability among countries, even within those integrated in the World Medical Association (WMA), according to the study by Moriaka and colleagues [17], based on a questionnaire survey of 13 national medical associations.

### 4.1. The main disadvantages of decentralized licensing

Decentralization has some advantages and some disadvantages. Among the disadvantages we point to the following:

(1) Differences in the requirements and documentation in admission to the register
  Each local council or college has the power to specify the documents and requirements for admission to practice. Hence, in a country with a number of provincial councils, a doctor might fulfill the requirements and thus becomes licensed in one province, while in the province next door she or he would not be able to work for lack of a required document.
(2) Differences in licensing fees and lack of financial control
  Most of these councils not only set the fee for registration and the periodic maintenance fee, but also set differences in fees among members, so that each local council ends up fees with great variation according to the type of member (doctors who are unemployed, in training, etc.). This sliding scale makes even more difficult a central control over what each council charges its members, and thus what in turn should be paid to the national association, affecting the finances of the national council.
(3) Growing separation from the national council
  Local councils and their members do not feel part of the national council. National councils do not take the initiative to develop programs like campaigns in defense of the profession or its members, therefore each local council must develop it on its own. Or, conversely, the national council may set up supplemental services directed at the local councils that are unnecessary and do not respond to local needs.
(4) Impoverishment of corporate identity
  In many instances, each local council or college uses a logo that is totally different from that of the national council. Similarly, they have different email accounts, domains and websites. As a result, there is an impoverishment of the image projected by the national councils, and a decline in the sense of unity on the part of local councils, and in consequence on the part of individual doctors, who may even be unaware of the existence of a council on the national level.
(5) Services unrelated to the medical profession
  The offer for members of services that have nothing to do with the medical profession is more common in local councils with obligatory membership. In such councils there may be expenditures difficult to justify, like, for example, gift baskets for the newborn babies of members. Such practices lead one to question whether the fees charged to members are more than what is needed in the councils and permit many of these organizations to embark on activities without relevance for the medical profession that distract for the purpose for which the councils were created.
(6) Differences in positions on health matters
  The national councils have serious problems in taking positions for any matter at the national level thus weakening the influence of the physicians' voice at institutional level. For example, if at the national level no position is taken in favor of alternative medicine, local councils may still decide to do so.
(7) Dubious national medical demographic statistics
  The national associations of doctors without centralized registers are forced to make a special effort to obtain the registers of each local college, especially since most often registration takes place in small offices with insufficient human, material and economic resources. Furthermore, the criteria for classification and the data categories involved may differ from local council to council, impeding the continuous updating of national information about doctors registered. The problem is not just in the transfer of data from the local college to the central office, but also in the local databases themselves, which may have errors due to a number of factors, such as classification and distribution in incorrect fields, lack of updating, errors in the manual data-processing, and the absence of internal computer controls. As a result, official national public statistics about doctors (such as the percentage of doctors who are unemployed, [3] practitioners, the numbers of doctors working in the private sphere, etc.) may be untrustworthy for countries with decentralized registers.
(8) Deficient mechanisms for the control of doctors who have lost their licenses
  If the principal reason to regulate the medical profession is the application of ways to penalize or take away the license of doctors with malpractice, then decentralization may be an obstacle. The mechanism is set in motion after a local council or court disqualifies one of the members, the general council is informed, and the general council in turn informs the rest of the councils. The problem lies in the manner by which local councils keep track of who is disqualified, and whether the database with their names is linked or not to the registration process. Those councils that are not computerized and do not have personnel technically qualified to connect the two databases may end up registering a doctor who has been de-licensed in another province. For in many cases the consultation of the malpractice database is manual, and thus whether the list of de-licensed doctors is checked or not depends on the human factor.

### 4.2. Advantages of decentralization of medical councils

In regard to the rest of the responsibilities entrusted to local councils or undertaken by them on their own initiative, decentralization seems to act to the doctors' advantage. For decentralized systems are better able to adapt to the idiosyncrasy of a particular region. The local directors of a local institution will know better the difficulties and conflicts faced by doctors in a given community, for which they can adopt suitable solutions. For the same reason they can resolve problems more rapidly when they come up. Doctors who are members of a local council are closer and more familiar with the bodies that represent them, which facilitates the participation in decisions and their application, resulting in an increased efficacy. By the same token, greater closeness means a greater identification of members with a council that is local, which results in a greater level of satisfaction of its members.

While decentralization has its advantages, they do not compensate the serious drawbacks of a decentralized national medical register, particularly in regard to the verification of the number and profile of the doctors registered, and of those professionals disqualified for malpractice by professional committees or judicial proceedings. It has been suggested that in some countries like Canada decentralization is the principal cause of a number of problems such as discrepancies in the provisional use of foreign medical licenses [19]. Focusing on the countries in which licensing is delegated to the regional, provincial o departmental level, it can be said that most of these local councils operate autonomously from the central council. The autonomy results in major discrepancies in the regulation of the medical profession within the same country, and this is the main drawback of decentralization.

Most of the problems identified in this study could be solved if the registration of doctors and their transfers between provinces were centralized in the central office. This would not require the physical presence of the professional at the general council, but simply that the local councils act as branches of the central office, and not as quasi-autonomous and independent entities in terms of the act of registration, as is the case now in the majority of the countries where this procedure is decentralized.

Another measure worth considering in order to address potential problems could be the creation of monitoring bodies (made up of tech professionals who are not council members) financed by the central council. Their mission would be to rotate through the different local centers training and performing the material tasks necessary for the correct functioning of those smaller councils as needed, as typically occurs in the private sector.

This study contributes to the knowledge of the medical organizations in the developed world. We detected problems that the literature had not yet considered, particularly the decentralization of the registry. These problems are important not only for the medical organizations, but they also cause problems to the medical system. We suggest some avenues for its solution.
